# The impact of physical activity on academic performance in children and adolescents: a meta-analysis

**DOI:** 10.3389/fpsyg.2026.1836195

**Published:** 2026-05-19

**Authors:** Zhihao Feng, Zhengyang Zhao, Ruobing Chen, Yongfeng Liu

**Affiliations:** School of Sports Training, Chengdu Sport University, Chengdu, Sichuan, China

**Keywords:** academic performance, adolescents, children, meta-analysis, physical activity

## Abstract

**Background:**

In contemporary education, the reduction of time allocated to physical activity under the pressure of heavy academic demands has become an increasingly prominent concern. At the same time, the World Health Organization has emphasized that moderate-to-vigorous physical activity is beneficial to students’ cognitive and academic development. However, existing findings on the effects of physical activity on the academic performance of children and adolescents remain inconsistent, and the optimal intervention characteristics for different academic domains have yet to be clearly established.

**Objective:**

This meta-analysis sought to systematically evaluate the effects of physical activity on the academic performance of children and adolescents aged 8–19 years and to explore whether pooled effects differed across intervention type, session duration, frequency, and intervention period for different academic outcomes, including mathematics, reading, spelling, and overall academic performance.

**Methods:**

A comprehensive search of PubMed, Web of Science, Embase, EBSCO, and the Cochrane Library was conducted up to January 10, 2026, to identify randomized controlled trials (RCTs) examining physical activity interventions and academic performance in the target population. Two reviewers independently carried out the screening process, extracted the relevant data, and evaluated methodological quality with the Cochrane Risk of Bias Tool. The meta-analysis was undertaken in Review Manager 5.3 under a random-effects model, and the results were reported as standardized mean differences (SMD) with 95% confidence intervals (CI). Sensitivity analyses were conducted, and funnel plots were interpreted descriptively where appropriate.

**Results:**

A total of 15 randomized controlled trials were included. Pooled analyses showed small but statistically significant effects of physical activity on mathematics performance (SMD = 0.06, 95% CI 0.01 to 0.10, *p* = 0.02) and reading performance (SMD = 0.10, 95% CI 0.04 to 0.16, *p* < 0.001). A statistically significant pooled effect was also observed for overall academic performance (SMD = 0.36, 95% CI 0.28 to 0.44, *p* < 0.001), although this finding was based on a relatively small number of studies. No statistically significant association was observed for spelling performance (SMD = 0.08, 95% CI − 0.03 to 0.19, *p* = 0.15), although the evidence base for this outcome was very limited. In exploratory subgroup analyses, some pooled effects reached statistical significance within specific subgroups; however, these findings were based on a small number of studies for several comparisons, reflected within-subgroup pooled estimates only, and should be interpreted cautiously.

**Conclusion:**

Physical activity interventions may be associated with small improvements in mathematics and reading. Evidence for overall academic performance appeared potentially favorable but remained limited, and evidence for spelling remained too sparse to support firm conclusions. The findings should be interpreted with caution given the broad age range of participants and the limited number of studies in several subgroup and outcome analyses.

**Systematic review registration:**

https://www.crd.york.ac.uk/PROSPERO/view/CRD420261294809, PROSPERO (CRD420261294809).

## 1 Introduction

In the contemporary educational landscape, the academic performance of children and adolescents remains a central concern for families, schools, and society at large. As curricular demands continue to intensify, many schools have curtailed time devoted to physical activity in order to preserve instructional hours, thereby posing challenges to the balance between students’ physical and mental well-being and their academic development. Current public health recommendations advise that children and adolescents engage in at least 60 min of moderate-to-vigorous physical activity each day, as this may support cognitive function, academic achievement, and mental well-being. Yet fewer than half of children and adolescents meet this recommendation, a phenomenon that has caused considerable concern among families and educators alike (Janssen and LeBlanc, 2010). In recent years, physical activity has come to be regarded not merely as a means of improving physical health but also as an important avenue for enhancing academic performance, mental well-being, and cognitive functioning.

A growing body of evidence suggests that regular physical activity is associated with improvements in cognitive functioning, attentional performance, and academic achievement among children and adolescents (Singh et al., 2019; Unterguggenberger, 2025). De Greeff et al. (2018) observed that both acute exercise and long-term exercise programs exert a moderate positive effect on children’s executive function and attention, as well as a small positive effect on academic performance. Hillman et al. (2014) found that children who engage in moderate-to-vigorous physical activity exhibit stronger neural activation during cognitive tasks, resulting in superior performance on mathematical reasoning tests. Singh et al. (2019) further demonstrated that physical activity interventions, particularly those integrated into classroom activities, may be beneficial for cognitive functioning, attention, and academic performance in children and adolescents.

Despite relatively consistent evidence for cognitive benefits, whether these advantages translate into measurable academic gains remains unsettled. In recent years, several investigations have examined the effects of physical activity interventions on academic performance in school settings. Some studies have found that integrating physical activity into subject instruction can significantly improve students’ academic outcomes (Resaland et al., 2016). Others, however, have failed to detect a significant positive effect (Tarp et al., 2016), while some have reported improvements in selected cognitive functions that did not translate into better academic performance. The findings, therefore, remain inconsistent. In addition, some scholars have suggested that physical activity may encroach upon study time and thereby diminish academic performance (Sallis et al., 1997).

In summary, these differences may be related to various factors such as experimental design, participant age, type and duration of physical activity, and methods of academic assessment. Although a few studies have attempted to integrate relevant literature, most have focused on specific groups and specific interventions, and the analysis of intervention types and subject impacts is still insufficient. Moreover, it is not yet clear what type of physical activity can promote the academic development of children and adolescents and whether there are subject differences in the impact of physical activity interventions on academic performance. Accordingly, the present meta-analysis of randomized controlled trials was undertaken to examine the association between physical activity interventions and academic performance in individuals aged 8–19 years and to identify intervention characteristics associated with different academic outcomes (Baker et al., 2024).

## 2 Methods

### 2.1 Protocol and registration

The review process adhered to the PRISMA guidelines and followed the recommendations outlined in the Cochrane Handbook for Systematic Reviews of Interventions (Moher et al., 2009; Chandler et al., 2019). Additionally, the review protocol was registered in advance in PROSPERO with the registration number CRD420261294809.

### 2.2 Search strategy

Relevant studies were identified through comprehensive searches of PubMed, Web of Science, Embase, EBSCO, and the Cochrane Library. Search terms were developed around three main concepts: physical activity interventions, children and adolescents, and academic performance. The search was conducted up to January 10, 2026. The PubMed search strategy is shown in Table 1, and the strategies for the remaining databases are provided in the Appendix.

**Table 1:** Summary of search terms.

Search terms	
#1	“Exercise”[Mesh]
#2	((((((((((((Physical Exercise[Title/Abstract]) OR (Isometric Exercise[Title/Abstract])) OR (Aerobic Exercise[Title/Abstract])) OR (Physical Activity[Title/Abstract])) OR (Active Break[Title/Abstract])) OR (Acute Exercise[Title/Abstract])) OR (aerobic training[Title/Abstract])) OR (fitness game[Title/Abstract])) OR (strength training[Title/Abstract])) OR (strength exercise[Title/Abstract])) OR (resistance exercise[Title/Abstract])) OR (sports activities[Title/Abstract])) OR (sport movement[Title/Abstract])
#3	#1 OR #2
#4	“Academic Performance”[Mesh]
#5	((((((((((((((((((Academic Test Score[Title/Abstract]) OR (Academic Test[Title/Abstract])) OR (Test Score[Title/Abstract])) OR (Test Performance[Title/Abstract])) OR (Educational Test[Title/Abstract])) OR (Educational Performance[Title/Abstract])) OR (academic outcome[Title/Abstract])) OR (academic achievement[Title/Abstract])) OR (academic success[Title/Abstract])) OR (academic attainment[Title/Abstract])) OR (school grades[Title/Abstract])) OR (scholastic performance[Title/Abstract])) OR (scholastic achievement[Title/Abstract])) OR (grade point average[Title/Abstract])) OR (standardized test score[Title/Abstract])) OR (learning[Title/Abstract])) OR (math[Title/Abstract])) OR (literacy[Title/Abstract])) OR (GPA[Title/Abstract])
#6	#4 OR #5
#7	“Adolescent”[Mesh]
#8	(((((Adolescence[Title/Abstract]) OR (Youth[Title/Abstract])) OR (Teen[Title/Abstract])) OR (Teenager[Title/Abstract])) OR (Student[Title/Abstract])) OR (child[Title/Abstract])
#9	#7 OR #8
#10	(RCT[Title/Abstract]) OR (“Randomized Controlled Trial” [Publication Type])
#11	#3 AND #6 AND #9 AND #10

Boolean operators (“AND” and “OR”) were used to combine these terms. The detailed search strategy is shown below: (“Physical Exercise” OR “Isometric Exercise” OR “Aerobic Exercise” OR “Physical Activity” OR “Active Break” OR “Acute Exercise” OR “aerobic training” OR “fitness game” OR “strength training” OR “strength exercise” OR “resistance exercise” OR “sports activities” OR “sport movement”) AND (“academic performance” OR “Academic Test Score” OR “Academic Test” OR “Test Score” OR “Test Performance” OR “Educational Test” OR “Educational Performance” OR “academic outcome” OR “academic achievement” OR “academic success” OR “academic attainment” OR “school grades” OR “scholastic performance” OR “scholastic achievement” OR “grade point average” OR “standardized test score” OR “learning” OR “math” OR “literacy” OR “GPA”) AND (“adolescents” OR “Youth” OR “Teen” OR “Teenager” OR “Student” OR “child”) AND (“randomized controlled trials” OR “RCT”). Table 1 presents the full search terms used in this review. All steps of the review process, including title and abstract screening, full-text evaluation, data extraction, and risk-of-bias assessment, were performed independently by two reviewers, with any disagreements settled through discussion or, when necessary, consultation with a third reviewer.

### 2.3 Inclusion and exclusion criteria

The eligibility criteria for this study were defined in accordance with the PICOS framework. Participants (P) were school-aged children and adolescents aged 8–19 years. This age range was predefined to reflect the target population commonly addressed in school-based physical activity and academic performance research. Interventions (I) included various forms of physical activity programs. Comparators (C) included regular physical education classes, no additional physical activity, or no exercise. Outcomes (O) were academic performance indicators, including mathematics, reading, spelling, and overall academic performance. Overall academic performance referred to global academic indicators, such as GPA, average subject grades, final examination scores, or other composite academic scores reported in the included studies. Study designs (S) were restricted to randomized controlled trials.

The following studies were excluded based on these criteria: studies involving adults, animals, or special populations; studies from which relevant data could not be extracted; duplicate publications; studies for which the full text was unavailable; and non-English publications, unpublished studies, reviews, conference abstracts, and other non-original research articles.

### 2.4 Data extraction

Study selection and data extraction were performed in accordance with the PRISMA guidelines. EndNote 21 software was used to remove duplicate records, and references retrieved from all databases were integrated into a unified database. Relevant information from eligible studies was extracted independently by two reviewers. Any discrepancies were resolved through discussion, and when necessary, consultation with a third reviewer. The extracted data mainly included study characteristics, participant information, intervention-related variables, and academic outcome measures. When relevant data were unavailable, we attempted to contact the corresponding authors for clarification. If the required data remained unavailable, the study was excluded.

The extracted information was organized in an Excel database, including the first author, year of publication, country, participant profile, intervention characteristics, and academic outcome measures. For quantitative synthesis, group-level data from the intervention and control groups, including sample sizes, means, and standard deviations, were entered into Review Manager 5.3 (Copenhagen: The Nordic Cochrane Centre, 2014).

### 2.5 Statistical analysis

Meta-analysis was performed in Review Manager 5.3 under a random-effects model (Cumpston et al., 2019), given the anticipated clinical and methodological heterogeneity across studies in participant characteristics, intervention content, intervention dose, and academic outcome measures. Because academic performance was assessed using different instruments across studies, pooled effects were expressed as standardized mean differences (SMD) with 95% confidence intervals (CI). Mathematics, reading, spelling, and overall academic performance were synthesized separately because they represent distinct academic domains.

Exploratory subgroup analyses were conducted according to intervention type, session duration, intervention frequency, and intervention period to examine potential differences in pooled effects across study characteristics. Because several subgroup comparisons were based on a small number of studies, these analyses were considered exploratory and were expected to have limited statistical power. Age was also considered a potentially important source of heterogeneity; however, a formal age-based moderator analysis was not feasible because the number of studies available for several outcomes was limited and the reporting of age characteristics was not sufficiently consistent across studies.

Statistical heterogeneity was assessed using the I^2^ statistic (Chandler et al., 2019). Leave-one-out sensitivity analyses were conducted to examine the robustness of the pooled estimates. Because fewer than 10 studies were available for each outcome, a formal assessment of publication bias was not considered sufficiently informative; therefore, funnel plots were interpreted descriptively only, and any apparent symmetry or asymmetry was treated with caution.

### 2.6 Methodological quality assessment

The methodological quality of the included studies was independently assessed by two reviewers using the Cochrane risk-of-bias tool in Review Manager 5.3. Any disagreements were resolved through discussion or consultation with a third reviewer. Seven domains were evaluated: random sequence generation, allocation concealment, blinding of participants and personnel, blinding of outcome assessment, incomplete outcome data, selective reporting, and other bias. Each domain was judged as low risk, unclear risk, or high risk, and these assessments were used to determine the overall risk of bias for each study.

## 3 Results

### 3.1 Study selection

**Figure 1:**
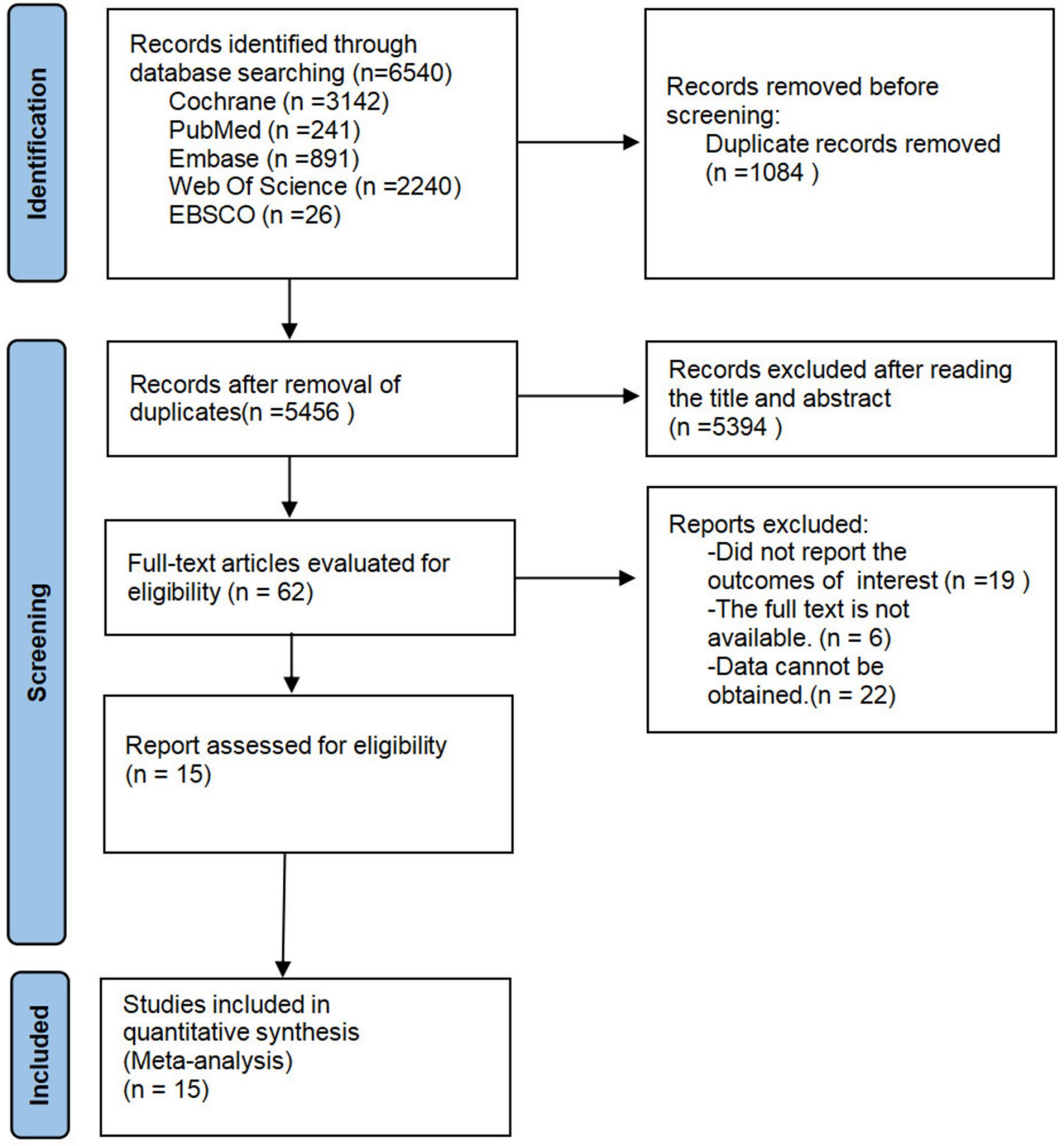
PRISMA flow diagram of the study process.

Figure 1 summarizes the study identification, screening, and inclusion process. A total of 6,540 records were identified across the databases, including 3,142 from the Cochrane Library, 241 from PubMed, 891 from Embase, 2,240 from Web of Science, and 26 from EBSCO. After removal of duplicates (*n* = 1,084), 5,456 records remained. Of these, 5,394 were excluded for failing to meet the inclusion criteria, including reviews (*n* = 387), non-RCTs (*n* = 101), reports, cross-sectional studies, and other article types (*n* = 390), as well as irrelevant studies (*n* = 4,516). Ultimately, 62 articles were retrieved for full-text review, of which 47 were excluded for the following reasons: no results reported (*n* = 19), full text unavailable (*n* = 6), and insufficient data (*n* = 22). In the end, 15 studies were included in the meta-analysis.

### 3.2 Study characteristics

The main characteristics of the included trials are summarized in Table 2, and the publication years ranged from 2009 to 2023. The sample size of the experimental group ranged from 9 to 1,275, and that of the control group ranged from 12 to 1,275. The mean age of participants ranged from 8 to 19 years. The intervention types in each study were PA or PAAL, with intervention frequencies ranging from once a week to five times a week, intervention durations ranging from 20 to 120 min, and intervention periods ranging from 4 to 156 weeks. Academic performance included four parts: mathematics, reading, spelling, and overall academic performance.

**Table 2:** Summary table of included studies.

Author (Year)	Country	Mean age (year)		Sample size		Intervention content (type)		Session duration, frequency, period		Outcome
		EG	CG	EG	CG	EG	CG	EG	CG	
Ardoy et al. (2014)	Spain	12.7	13.8	23	18	Increase the frequency and intensity of physical education classes (PA)	Regular physical education class	55 min, 4 times/week, 4 months	55 min,2 times/week, 4 months	Mathematics, Reading
De Bruijn et al. (2020)	Netherlands	9.24		221	430	Aerobic activity (PA)	Regular physical education class	30 min, 4 times/week, 10 weeks	No additional PA intervention	Mathematics, Reading, Spelling
Donnelly et al. (2017)	USA	8.1		244	207	Physically active academic lessons (PAAL)	Traditional pedagogy	20 min, 3 times/week, 3 years	No additional PA intervention	Mathematics, Reading, Spelling
Hraste et al. (2018)	Croatia	10.36		19	17	Geometric teaching integrated with physical activities (PAAL)	Traditional pedagogy	45 min, 1 time/week, 4 weeks	No additional PA intervention	Mathematics
Solberg et al. (2021b)	Norway	14		491	483	PAL/DWBH (PAAL)	Regular physical education class	40 min, 3 times/week 9 months	No additional PA intervention	Mathematics, Reading
Joubert et al. (2017)	USA	19		9	12	Combining cycling with classroom learning (PA)	Traditional sitting posture for listening to lectures	50 min, 3 times/week, 12 weeks	No additional PA intervention	Overall academic performance
Lubans et al. (2018)	Australia	12.92		584	580	Activity and Motivation in Physical Education (PA)	Regular physical education class teaching	30 min, 2 times/week, 2.5 years	No additional PA intervention	Mathematics
Melero-Cañas et al. (2021)	Spain	14.63		113	37	Game-based and Responsibility-integrated Physical Education Teaching (PAAL)	Regular physical education class	55 min, 2 times/week, 9 months	No additional PA intervention	Overall academic performance
Mullender-Wijnsma et al. (2016)	Netherlands	8.1		249	250	Integrating moderate-to-high-intensity physical activities into mathematics and language teaching (PAAL)	Traditional pedagogy	20 min, 3 times/week, 2 years	No additional PA intervention	Mathematics, Reading, Spelling
Peternelj et al. (2009)	Slovenia	11		68	66	Regular physical education classes combined with additional physical activities (PA)	Regular physical education class	30 min, 3 times/week, 8 months	No additional PA intervention	Overall academic performance
Riley et al. (2016)	Australia	11.13		142	98	Mathematics teaching integrated with physical activities (PAAL)	Traditional pedagogy	60 min, 3 times/week, 6 weeks	No additional PA intervention	Mathematics
Solberg et al. (2021a,b)	Norway	14		1,275	1,275	Multi-component school-based physical activity intervention (PA)	Traditional pedagogy	50–60 min, 2 times/week, 9 months	No additional PA intervention	Mathematics, Reading
Takehara et al. (2021)	Mongolia	9.7		1,069	1,032	Structured physical activities are arranged in the school curriculum (PAAL)	Traditional pedagogy	25 min, 2 times/week, 10 weeks	No additional PA intervention	Overall academic performance
Vetter et al. (2018)	Australia	8.4		170	170	Integrating physical activities into mathematics classes (PAAL)	Traditional pedagogy	30 min, 3 times/week, 6 weeks	No additional PA intervention	Mathematics
Wang et al. (2023)	China	9.22		1,012	1,020	After-school physical activities (PA)	Arrange your after-school time freely	120 min, 5 times/week, 9 months	No additional PA intervention	Mathematics

EG experimental group, CG control group, PA traditional physical activity, PAAL physical activity integrated into the academic curriculum.

### 3.3 Risk of bias

**Figure 2:**
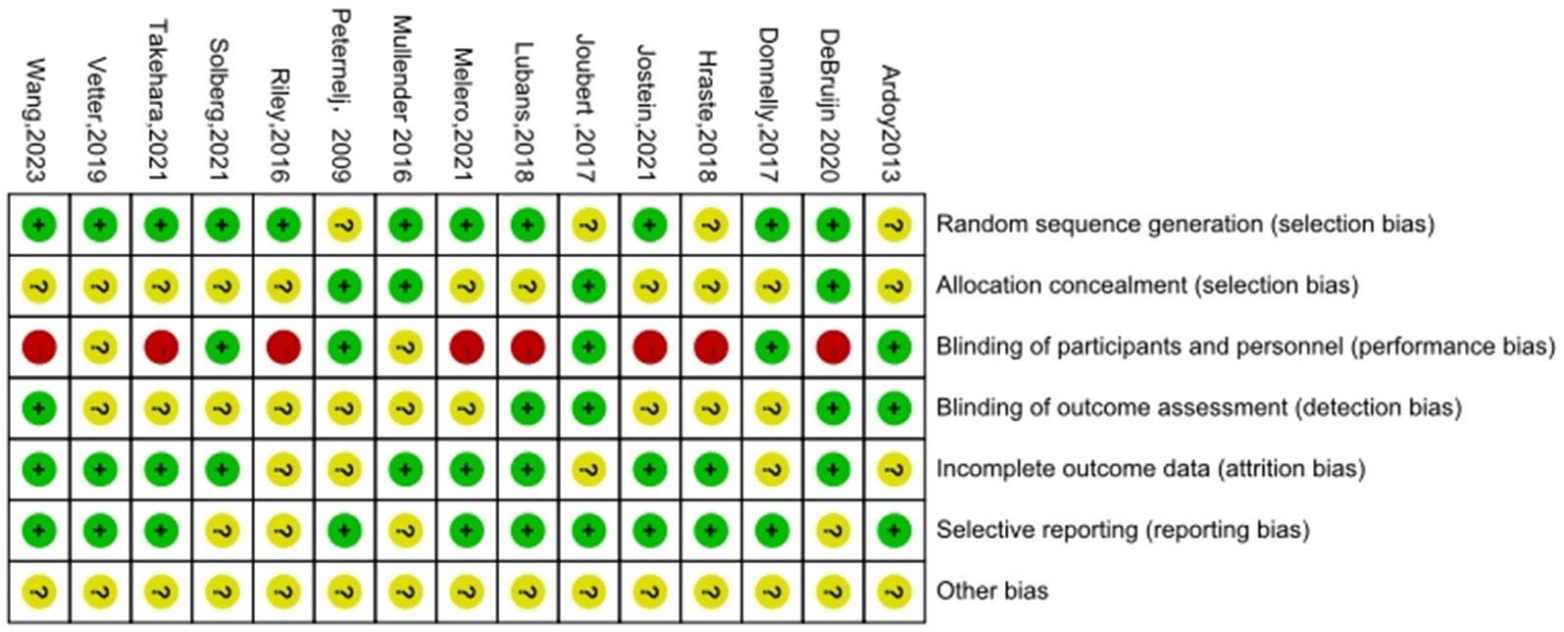
Methodological quality of included studies.

**Figure 3:**
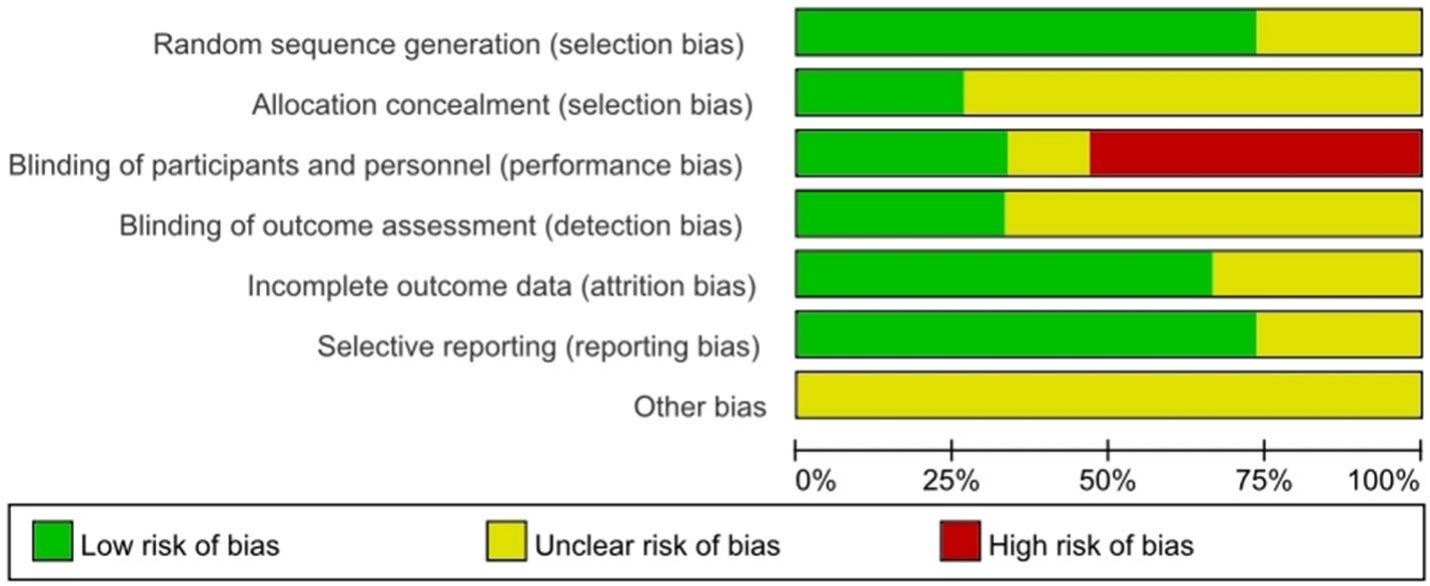
The distribution of the methodological quality of included studies.

For the generation of random sequences, 11 studies (Lubans et al., 2018; Mullender-Wijnsma et al., 2016; Donnelly et al., 2017; Vetter et al., 2018; Riley et al., 2016; Takehara et al., 2021; Melero-Cañas et al., 2021; De Bruijn et al., 2020; Solberg et al., 2021a; Wang et al., 2023; Solberg et al., 2021b) were rated as low risk, and 4 studies (Hraste et al., 2018; Ardoy et al., 2014; Joubert et al., 2017; Peternelj et al., 2009) were rated as having unclear risk. Regarding the allocation bias, 4 studies (Mullender-Wijnsma et al., 2016; De Bruijn et al., 2020; Joubert et al., 2017; Peternelj et al., 2009) were rated as low risk, and 11 studies (Lubans et al., 2018; Donnelly et al., 2017; Vetter et al., 2018; Riley et al., 2016; Takehara et al., 2021; Melero-Cañas et al., 2021; Solberg et al., 2021a; Wang et al., 2023; Solberg et al., 2021b; Hraste et al., 2018; Ardoy et al., 2014) were rated as having unclear risk. Regarding the performance bias, 5 studies (Donnelly et al., 2017; Solberg et al., 2021a; Ardoy et al., 2014; Joubert et al., 2017; Peternelj et al., 2009) were rated as low risk, 2 studies (Mullender-Wijnsma et al., 2016; Vetter et al., 2018) were rated as having unclear risk, and 8 studies (Lubans et al., 2018; Riley et al., 2016; Takehara et al., 2021; Melero-Cañas et al., 2021; De Bruijn et al., 2020; Wang et al., 2023; Solberg et al., 2021b; Hraste et al., 2018) were rated as high risk. Regarding the detection bias, 5 studies (Lubans et al., 2018; De Bruijn et al., 2020; Wang et al., 2023; Ardoy et al., 2014; Joubert et al., 2017) were rated as low risk, and 10 studies (Mullender-Wijnsma et al., 2016; Donnelly et al., 2017; Vetter et al., 2018; Riley et al., 2016; Takehara et al., 2021; Melero-Cañas et al., 2021; Solberg et al., 2021a; Solberg et al., 2021b; Hraste et al., 2018; Peternelj et al., 2009) were rated as having unclear risk. For the attrition bias, 10 studies (Lubans et al., 2018; Mullender-Wijnsma et al., 2016; Vetter et al., 2018; Takehara et al., 2021; Melero-Cañas et al., 2021; De Bruijn et al., 2020; Solberg et al., 2021a; Wang et al., 2023; Solberg et al., 2021b; Hraste et al., 2018) were rated as low risk, and 5 studies (Donnelly et al., 2017; Riley et al., 2016; Ardoy et al., 2014; Joubert et al., 2017; Peternelj et al., 2009) were rated as having unclear risk. Regarding the reporting bias, 11 studies (Lubans et al., 2018; Donnelly et al., 2017; Vetter et al., 2018; Takehara et al., 2021; Melero-Cañas et al., 2021; Wang et al., 2023; Solberg et al., 2021b; Hraste et al., 2018; Ardoy et al., 2014; Joubert et al., 2017; Peternelj et al., 2009) were rated as low risk, and 4 studies (Mullender-Wijnsma et al., 2016; Riley et al., 2016; De Bruijn et al., 2020; Solberg et al., 2021a) were rated as having unclear risk. Regarding other biases, 15 studies (Lubans et al., 2018; Mullender-Wijnsma et al., 2016; Donnelly et al., 2017; Vetter et al., 2018; Riley et al., 2016; Takehara et al., 2021; Melero-Cañas et al., 2021; De Bruijn et al., 2020; Solberg et al., 2021a; Wang et al., 2023; Solberg et al., 2021b; Hraste et al., 2018; Ardoy et al., 2014; Joubert et al., 2017; Peternelj et al., 2009) were rated as having unclear risk. Overall, most studies showed low or unclear risk in sequence generation and attrition domains, whereas performance bias was more frequently rated as high risk because blinding participants in physical activity interventions is inherently difficult. Detailed results of the risk-of-bias assessment are shown in Figures 2, 3.

### 3.4 Meta-analysis

Exploratory subgroup analyses were conducted to examine factors that might influence the association between physical activity and academic performance. The studies included in the analysis categorized interventions into two types, traditional physical activity (PA) and physical activity combined with academic curriculum (PAAL). The duration of intervention was divided into “less than or equal to 30 minutes” and “more than 30 minutes.” The frequency of intervention was classified as “less than or equal to 3 times/week” and “more than 3 times/week.” The intervention period was divided into “less than or equal to 12 weeks” and “more than 12 weeks”. Academic performance was divided into four indicators: mathematics, reading, spelling, and overall academic performance. These subgroup classifications were defined with reference to previous research and school physical activity guidelines (Jeppesen et al., 2024; Xu et al., 2023; He et al., 2025). In addition, these subgroup analyses were exploratory in nature, reflected differences in within-subgroup pooled estimates, and should not be interpreted as definitive evidence of statistically significant differences between subgroups. Because several subgroup comparisons were based on a small number of studies, their statistical power was limited, and the corresponding findings should be interpreted cautiously.

#### 3.4.1 The impact of physical activity on mathematics

**Figure 4:**
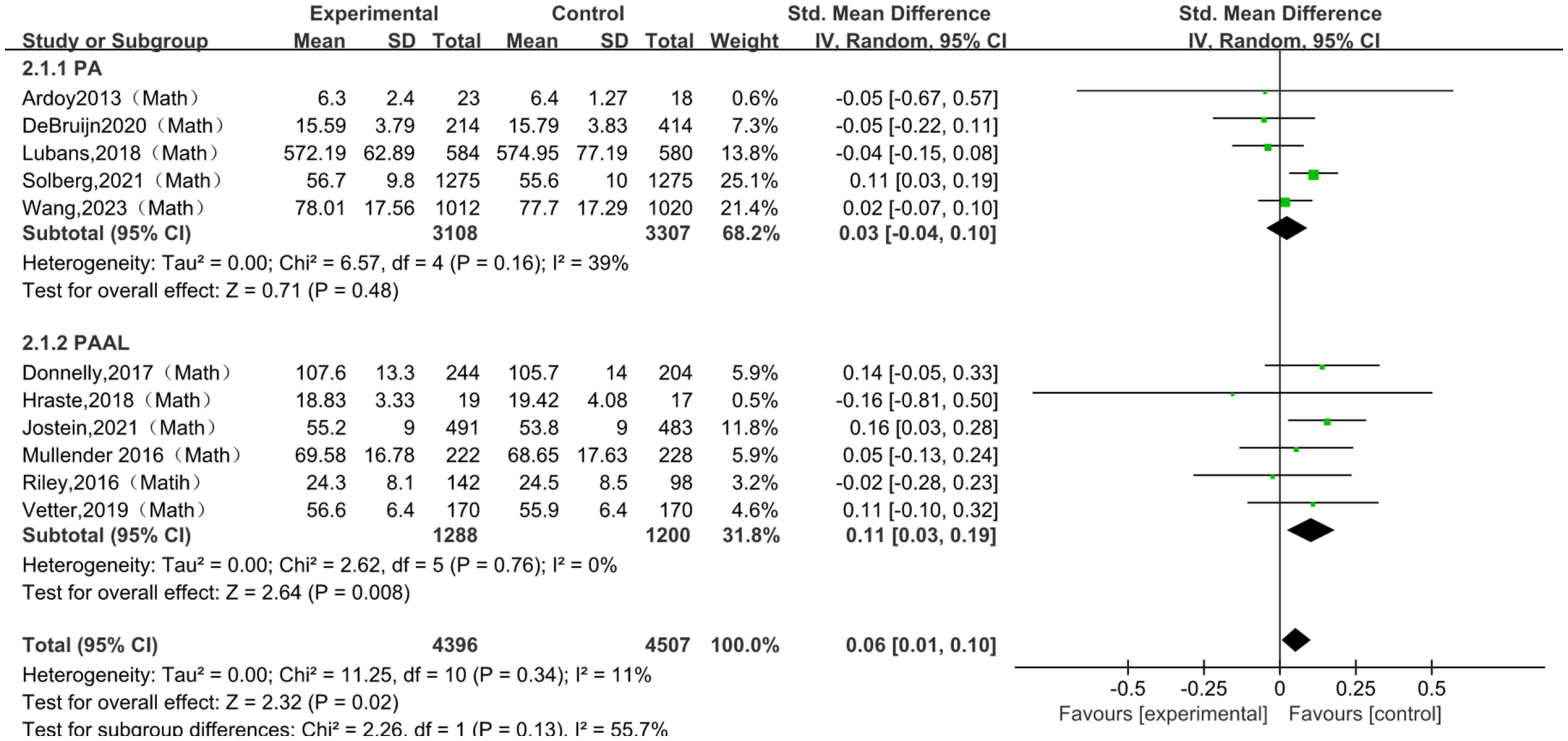
The subgroup analysis of the effect of physical activity intervention type on math.

A total of 11 studies (Lubans et al., 2018; Mullender-Wijnsma et al., 2016; Donnelly et al., 2017; Vetter et al., 2018; Riley et al., 2016; De Bruijn et al., 2020; Solberg et al., 2021a; Wang et al., 2023; Solberg et al., 2021b; Hraste et al., 2018; Ardoy et al., 2014) were included in the meta-analysis of mathematics performance. The pooled analysis showed a small but statistically significant association between physical activity and mathematics performance (SMD = 0.06, 95% CI 0.01 to 0.10, *p* = 0.02; I^2^ = 11%). To investigate possible variation in intervention effects, subgroup analyses were undertaken by intervention type, session duration, frequency, and intervention period. The subgroup results are presented in Figure 4 and Table 3. Significant pooled effects were observed in the PAAL subgroup (SMD = 0.11, 95% CI 0.03 to 0.19, *p* = 0.008, I^2^ = 0%), in the subgroup with session durations longer than 30 min (SMD = 0.08, 95% CI 0.03 to 0.13, *p* = 0.003, I^2^ = 5%), in the subgroup with an intervention frequency of no more than three times per week (SMD = 0.08, 95% CI 0.02 to 0.13, *p* = 0.005, I^2^ = 10%), and in the subgroup with intervention periods longer than 12 weeks (SMD = 0.07, 95% CI 0.01 to 0.12, *p* = 0.020, I^2^ = 28%).

**Table 3:** The subgroup analysis of the effect of physical activity intervention type on math.

Subgroup	Subgroup criteria	Literature quantity	I^2^	SMD (95% CI)	*p*
Type	PA	5	39%	0.03[−0.04,0.10]	0.480
	PAAL	6	0%	0.11[0.03,0.19]	0.008
Session duration	≤30 min	5	2%	0.02[−0.06,0.09]	0.660
	>30 min	6	5%	0.08[0.03,0.13]	0.005
frequency	≤3 times/week	8	10%	0.08[0.02,0.13]	0.005
	>3 times/week	3	0%	0.00[−0.07,0.08]	0.960
Intervention period	≤12 weeks	4	0%	0.00[−0.12,0.11]	0.940
	>12 weeks	7	28%	0.07[0.01,0.12]	0.020

PA, traditional physical activity; PAAL, physical activity integrated into the academic curriculum.

#### 3.4.2 The impact of physical activity on reading

**Figure 5:**
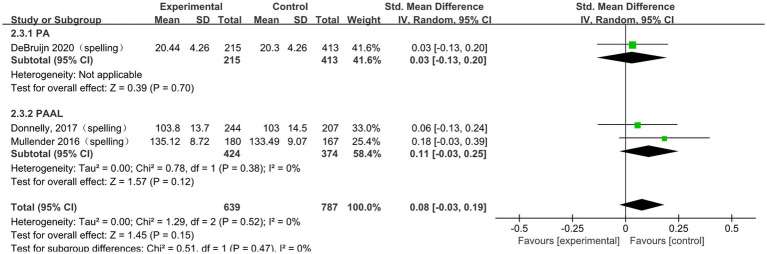
The subgroup analysis of the effect of physical activity intervention type on reading.

A total of 6 studies (Mullender-Wijnsma et al., 2016; Donnelly et al., 2017; De Bruijn et al., 2020; Solberg et al., 2021a; Solberg et al., 2021b; Ardoy et al., 2014) were included in the meta-analysis of reading performance. The pooled analysis showed a small but statistically significant association between physical activity and reading performance (SMD = 0.10, 95% CI 0.04 to 0.16, *p* < 0.001; I^2^ = 0%). To further explore potential differences in intervention effects, subgroup analyses were conducted according to intervention type, session duration, frequency, and intervention period. The subgroup results are presented in Figure 5 and Table 4. Significant pooled effects were observed in the PA subgroup (SMD = 0.11, 95% CI 0.04 to 0.18, *p* = 0.003; I^2^ = 0%), in the subgroup with session durations longer than 30 min (SMD = 0.12, 95% CI 0.05 to 0.19, *p* = 0.0004), in the subgroup with an intervention frequency of no more than three times per week (SMD = 0.11, 95% CI 0.04 to 0.17, *p* = 0.0007; I^2^ = 0%), and in the subgroup with intervention periods longer than 12 weeks (SMD = 0.10, 95% CI 0.04 to 0.16, *p* = 0.0008; I^2^ = 0%). Overall, subgroup analyses suggested that the pooled effects on reading performance varied across different intervention characteristics.

**Table 4:** The subgroup analysis of the effect of physical activity intervention type on reading.

Subgroup	Subgroup criteria	Literature quantity	I^2^	SMD (95% CI)	*p*
Type	PA	3	0%	0.11[0.04, 0.18]	0.003
	PAAL	3	0%	0.08[−0.01, 0.18]	0.080
Session duration	≤30 min	3	0%	0.05[−0.06, 0.16]	0.370
	>30 min	3	0%	0.12[0.05, 0.19]	0.0004
frequency	≤3 times/week	4	0%	0.11[0.04, 0.17]	0.0007
	>3 times/week	2	0%	0.07[−0.09, 0.23]	0.420
Intervention period	≤12 weeks	1	/	0.08[−0.08, 0.25]	0.320
	>12 weeks	5	0%	0.10[0.04, 0.16]	0.0008

PA, traditional physical activity; PAAL, physical activity integrated into the academic curriculum.

#### 3.4.3 The impact of physical activity on spelling

**Figure 6:**
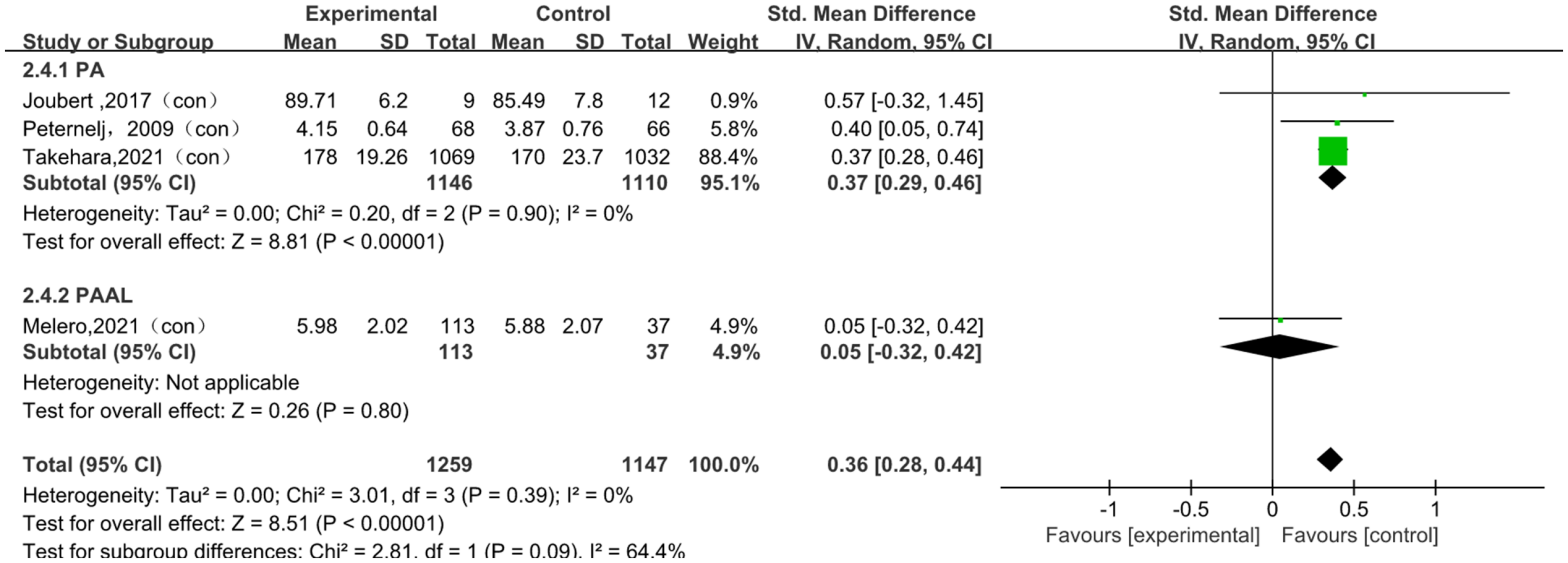
The subgroup analysis of the effect of physical activity intervention type on spelling.

A total of 3 studies (Mullender-Wijnsma et al., 2016; Donnelly et al., 2017; De Bruijn et al., 2020) were included in the meta-analysis of spelling performance. The pooled analysis did not show a statistically significant association between physical activity and spelling performance (SMD = 0.08, 95% CI − 0.03 to 0.19, *p* = 0.15). To further explore potential differences in intervention effects, subgroup analyses were conducted according to intervention type, session duration, frequency, and intervention period. The subgroup results are presented in Figure 6 and Table 5. No statistically significant pooled effects were identified in these exploratory subgroup analyses; however, interpretation was limited by the very small number of included studies and the correspondingly low statistical power.

**Table 5:** The subgroup analysis of the effect of physical activity intervention type on spelling.

Subgroup	Subgroup criteria	Literature quantity	I^2^	SMD (95% CI)	*p*
Type	PA	1	/	0.03[−0.13, 0.20]	0.700
	PAAL	2	0%	0.11[−0.03, 0.25]	0.120
Session duration	≤30 min	3	0%	0.08[−0.03, 0.19]	0.150
	>30 min	0	/	/	/
frequency	≤3 times/week	2	0%	0.11[−0.03, 0.25]	0.120
	>3 times/week	1	/	0.03[−0.13, 0.20]	0.700
Intervention period	≤12 weeks	1	/	0.03[−0.13, 0.20]	0.700
	>12 weeks	2	0%	0.11[−0.03, 0.25]	0.120

PA, traditional physical activity; PAAL, physical activity integrated into the academic curriculum.

#### 3.4.4 The impact of physical activity on overall academic performance

**Figure 7:**
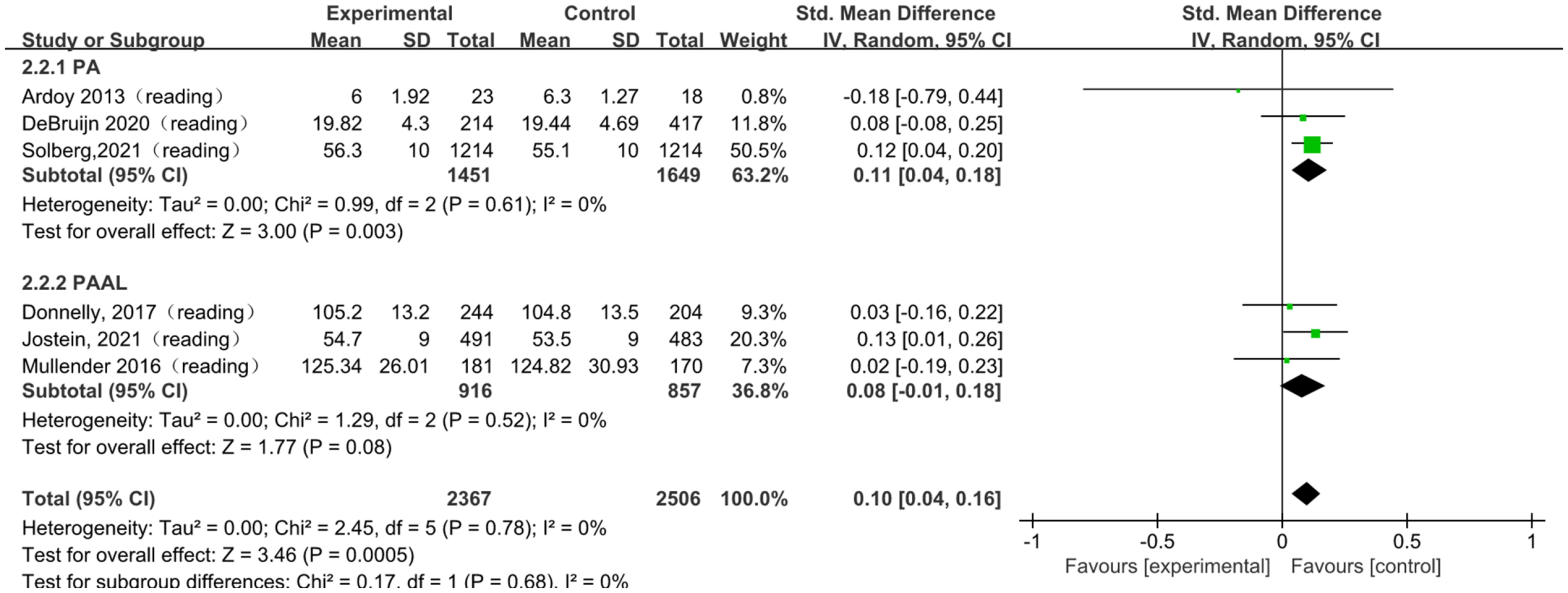
The Subgroup analysis of the effect of physical activity intervention type on the overall academic performance.

A total of 4 studies (Takehara et al., 2021; Melero-Cañas et al., 2021; Joubert et al., 2017; Peternelj et al., 2009) were included in the meta-analysis of overall academic performance. The pooled analysis showed a statistically significant association between physical activity and overall academic performance (SMD = 0.36, 95% CI 0.28 to 0.44, *p* < 0.001). To delve deeper into potential differences in the effects of interventions, subgroup analyses were conducted based on intervention type, session duration, frequency, and intervention period. The subgroup results are presented in Figure 7 and Table 6. Significant pooled effects were observed in the PA subgroup (SMD = 0.37, 95% CI 0.29 to 0.46, *p* < 0.001; I^2^ = 0%), in the subgroup with session durations of 30 min or less (SMD = 0.37, 95% CI 0.29 to 0.46, *p* < 0.001; I^2^ = 0%), in the subgroup with an intervention frequency of no more than three times per week (SMD = 0.36, 95% CI 0.28 to 0.44, *p* < 0.001; I^2^ = 0%), and in the subgroup with intervention periods of 12 weeks or less (SMD = 0.37, 95% CI 0.29 to 0.46, *p* < 0.0001; I^2^ = 0%). Overall, subgroup analyses suggested that the pooled effects on overall academic performance varied across different intervention characteristics. These findings for overall academic performance were based on a very small number of studies, which limited statistical power and reduced the stability of subgroup-specific estimates; therefore, they should be interpreted with particular caution.

**Table 6:** The Subgroup analysis of the effect of physical activity intervention type on the overall academic performance.

Subgroup	Subgroup criteria	Literature quantity	I^2^	SMD (95% CI)	*p*
Type	PA	3	0%	0.37[0.29, 0.46]	<0.001
	PAAL	1	/	0.05[−0.32, 0.42]	0.800
Session duration	≤30 min	2	0%	0.37[0.29, 0.46]	<0.001
	>30 min	2	10%	0.14[−0.25, 0.54]	0.470
Frequency	≤3 times/week	4	0%	0.36[0.28, 0.44]	<0.001
	>3 times/week	0	/	/	/
Intervention period	≤12 weeks	2	0%	0.37[0.29, 0.46]	<0.001
	>12 weeks	2	45%	0.23[−0.11, 0.57]	0.180

PA, traditional physical activity; PAAL, physical activity integrated into the academic curriculum.

### 3.5 Sensitivity analysis

A leave-one-out sensitivity analysis was conducted by removing each study in turn to assess its influence on the pooled effect estimates. The results were generally stable, although the small number of studies for some outcomes limited interpretability. In particular, after exclusion of the study involving participants at the upper end of the included age range (Joubert et al., 2017), the pooled effect for overall academic performance remained statistically significant, and the direction and magnitude of the effect did not materially change, suggesting that the main finding was not driven solely by this sample.

### 3.6 Publication bias

**Figure 8:**
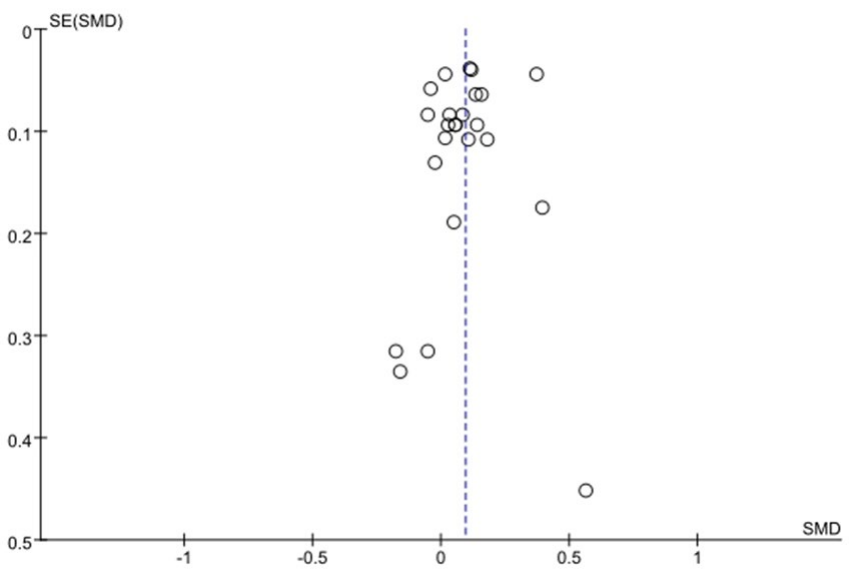
Funnel plot of publication bias risk.

The plot displayed the standardized mean difference (SMD) along the horizontal axis and the standard error of the standardized mean difference (SE [SMD]) along the vertical axis. Upon visual examination, it was observed that the effect sizes from the studies included in the analysis were distributed symmetrically around the combined effect estimate. The majority of studies were situated in the upper section of the funnel plot, indicating a higher level of precision, while those with lower precision were more scattered in the lower section. In general, the funnel plot appeared to exhibit a symmetrical pattern. However, due to the limited number of studies (less than 10) available for each specific outcome analysis, it was challenging to interpret any potential asymmetry in the funnel plot, and determining the presence of publication bias was not feasible (Sterne and Harbord, 2004) (Figure 8).

## 4 Discussion

### 4.1 Main results

By synthesizing 15 randomized controlled trials, the present review examined how physical activity interventions were related to academic outcomes in children and adolescents. The pooled results suggested small improvements in mathematics, reading, and overall academic performance, whereas no statistically significant pooled effect was observed for spelling. However, the strength of evidence differed across outcomes. Mathematics was informed by a relatively larger number of studies, but the pooled effect size remained small and should therefore be interpreted cautiously. Reading was based on fewer studies, which limits confidence in the stability of the pooled estimate. Overall academic performance showed a statistically significant pooled effect, but this outcome was informed by only a small number of studies and should therefore also be interpreted with caution. One possible reason why the pooled effect for overall academic performance appeared larger than those for mathematics and reading is that overall academic performance reflects broader and more aggregated academic indicators, such as GPA, composite grades, or final examination scores, rather than performance in a single subject domain. Such outcomes may be more sensitive to improvements in general classroom attention, learning motivation, emotional regulation, and self-efficacy, which can be influenced by physical activity. In addition, because the pooled estimate for overall academic performance was based on a relatively small number of studies, the corresponding effect size may be less stable. For spelling, the evidence base was too sparse to support firm conclusions and should be regarded as exploratory. These findings should also be interpreted in light of the broad developmental span of the included samples; accordingly, the pooled estimates are better regarded as broad cross-developmental summaries rather than stage-specific effects. In exploratory subgroup analyses, some pooled effects reached statistical significance within specific subgroups; however, these findings were based on relatively small numbers of studies for several comparisons, which limited statistical power and reduced confidence in the stability of the subgroup-specific estimates. Therefore, they should not be interpreted as definitive evidence of superiority between subgroups and are better viewed as exploratory and hypothesis-generating.

### 4.2 The impact of different types of physical activities on academic performance

This review synthesized 15 randomized controlled trials involving children and adolescents aged 8–19 years to examine whether physical activity interventions were associated with academic performance and whether pooled effects varied across intervention characteristics. Exploratory subgroup analyses suggested that different types of physical activity may be associated with different pooled patterns across academic outcomes. Small pooled effects on mathematics were observed mainly in the PAAL subgroup, whereas pooled effects on reading and overall academic performance were observed mainly in the PA subgroup. This pattern is broadly consistent with previous reviews (Loturco et al., 2022; Cipriano et al., 2024; Li et al., 2023).

One possible interpretation is that PAAL may be particularly relevant to mathematics-related outcomes. The learning of mathematics is influenced by various factors such as the processing speed of the brain, memory capacity, and intelligence (Geary, 2011). The PAAL curriculum may enhance students’ concentration, working memory, and executive functioning through physically active learning experiences (Norris et al., 2020), which is a prerequisite for complex mathematical thinking. Moreover, physical activities may increase students’ classroom concentration time, reduce distracting behaviors in class, and carry the concentration effect into subsequent mathematics teaching. Such activities may also increase students’ learning motivation and classroom participation (Gonzalez-Perez et al., 2025; González-Pérez et al., 2025). Additionally, through physical activities, students’ understanding of geometric shapes may be enhanced and their comprehension of abstract formulas may be deepened. Li′s meta-analysis indicates that the PAAL intervention has a particularly significant effect on improving mathematics performance (Li et al., 2023). Beck et al. stated that physical activities may improve short-term memory and classroom concentration, thereby enhancing academic performance (Beck et al., 2016).

Reading development relies more strongly on cumulative language exposure and vocabulary growth, which may mean that the contribution of physical activity is comparatively indirect. Overall academic performance, which is composed of outcomes from multiple subjects, may be more sensitive than single-subject outcomes to broad cognitive and non-cognitive changes, including executive functioning, classroom attention, emotional regulation, academic stress, and self-efficacy. The beneficial effects of traditional PA on reading and overall academic performance may therefore operate through improvements in these more general learning-related capacities (Visier-Alfonso et al., 2022).

Evidence regarding spelling was particularly limited. Only three studies reported spelling outcomes, and this sparse evidence base does not support firm conclusions; therefore, the corresponding findings should be interpreted as exploratory. One possible explanation is that spelling is a fundamental language function that relies heavily on linguistic memory and motor memory, which differ from the cognitive functions improved by physical activity. Spelling depends less on executive function compared to more complex cognitive tasks such as mathematics and reading. Moreover, spelling requires language input and writing practice, which physical activity cannot directly facilitate.

### 4.3 The impact of physical activity duration on academic performance

This study explored the relationship between the duration of a single physical activity session and academic performance in children and adolescents, revealing different subgroup patterns across outcomes. Statistically significant pooled effects for mathematics and reading were observed in subgroups with session durations longer than 30 min, whereas for overall academic performance, a statistically significant pooled effect was observed among studies with session durations of 30 min or less. These patterns were not consistent across outcomes and should therefore be interpreted cautiously.

One possible explanation is that physical activity sessions lasting longer than 30 min may be more likely to activate brain regions related to executive functioning, such as the prefrontal cortex, thereby supporting academic outcomes that rely on concentration and logical reasoning (Lv et al., 2024). Nevertheless, these explanations remain speculative. Hillman et al. pointed out that moderate-intensity aerobic exercise lasting over 30 min can significantly enhance children’s brain electrical activity and improve their performance in cognitive tasks (Hillman et al., 2009). Additionally, De Greeff’s et al. (2018) meta-analysis also found that longer-duration interventions are more likely to improve children’s executive functions and attention, which are the foundations of mathematical and reading abilities. However, in terms of overall academic performance, physical activities lasting 30 min or less appeared to show more favorable pooled effects. This might be related to the fact that overall academic performance encompasses a wider range of subjects and requires a more comprehensive set of cognitive functions. Physical activities lasting less than 30 min may help support students’ concentration, emotional state, and learning enthusiasm while requiring less energy expenditure. In addition, shorter and more fragmented activities may be easier to integrate into daily class schedules and may reduce the likelihood of fatigue associated with prolonged exercise (Atakan and Atakan, 2024; Drollette et al., 2025).

### 4.4 The impact of physical activity frequency on academic performance

Exploratory subgroup analyses suggested that pooled effects for mathematics, reading, and overall academic performance were more often observed in studies with intervention frequencies of no more than three times per week; however, this pattern should be interpreted cautiously and should not be regarded as evidence of an optimal prescription. One possible explanation is that moderate-frequency interventions may strike a better balance between cognitive stimulation and fatigue (Wang and Guo, 2022). On the one hand, high-frequency physical activity may lead to physical and mental fatigue, distraction, and occupation of subject teaching time for students, thereby reducing the positive effect of physical activity on cognitive function and academic performance to a certain extent (Bodensohn et al., 2024). On the other hand, too low an intervention frequency makes it difficult to reach the exercise intensity required to improve cognitive function and neural stimulation (Liu et al., 2025). However, these findings for overall academic performance were based on very few studies and should be interpreted with caution.

### 4.5 The impact of physical activity intervention period on academic performance

This study found that the intervention period of physical activity showed differences in various academic performance indicators. For mathematics and reading, statistically significant pooled effects were observed in subgroups with intervention periods longer than 12 weeks. Improvements in mathematics and reading may be related to enhanced executive functioning, and substantial changes in executive functioning may be more likely to emerge following longer-term and regular physical activity, potentially through cumulative neural and cognitive adaptations (Zheng et al., 2019; Valkenborghs et al., 2019). At the same time, the transfer effect of physical activity on subject learning has a lag, and it takes a relatively long time to internalize and apply it to subject learning (Owen et al., 2023). For overall academic performance, a statistically significant pooled effect was observed in studies with intervention periods of 12 weeks or less. One possible explanation for the relatively larger pooled effect observed for overall academic performance is that this outcome is more comprehensive and may be more strongly influenced by non-cognitive factors such as classroom concentration, emotional state, and learning enthusiasm (Maiztegi-Kortabarria et al., 2024). In the early stage of physical activity, students’ high learning enthusiasm and classroom activity levels were generated due to the novelty of physical activity, which temporarily improved overall academic performance. However, as the intervention period extended, students gradually felt fatigued, and the positive effects of physical activity gradually decreased. Moreover, short-term physical activity can increase blood flow and dopamine secretion, temporarily enhancing the cognitive functions required for comprehensive examinations and thereby improving short-term overall academic performance (Naito et al., 2024; Berse et al., 2015). These interpretations, however, remain tentative.

## 5 Limitations and deficiencies

Several limitations should be acknowledged. First, several subgroup analyses, particularly those for spelling and overall academic performance, were based on a small number of studies, which limited statistical power and reduced the stability of subgroup-specific estimates. Second, these subgroup analyses were exploratory in nature and reflected within-subgroup pooled effects only; therefore, they should not be interpreted as formal evidence of statistically significant differences between subgroups and should instead be viewed as hypothesis-generating. Third, the included studies varied in participant characteristics, intervention content, outcome measures, and study design, including several cluster-randomized trials, which may have introduced methodological heterogeneity. Fourth, the included population covered a broad developmental span (8–19 years), which may have reduced population homogeneity because younger children and older adolescents may differ in cognitive development, school context, and responsiveness to physical activity interventions. Moreover, a robust age-based moderator analysis was not feasible due to the limited number of studies for several outcomes and inconsistent reporting of age characteristics. Finally, insufficient reporting in the original studies prevented a more detailed examination of potentially important moderators such as exercise intensity.

## 6 Conclusion

This meta-analysis suggests that physical activity may be associated with small benefits in mathematics, although the practical significance of this effect should be interpreted cautiously. For reading, the pooled findings appear potentially favorable but are based on a relatively limited number of studies and therefore warrant additional caution. For overall academic performance, the observed pooled effect may be encouraging, but the evidence base remains limited by the small number of available studies. Evidence for spelling remains too sparse to support firm conclusions and should be regarded as exploratory. Moreover, because the included studies covered a broad developmental span, the pooled findings should not be interpreted as evidence that the observed effects are uniform across developmental stages. Exploratory subgroup analyses suggested different pooled patterns according to intervention type, session duration, frequency, and intervention period; however, these patterns were not uniform across outcomes and were based on a limited number of studies for several comparisons. Therefore, these subgroup-specific findings should be interpreted cautiously and should not be regarded as confirmatory evidence of superiority for any particular intervention characteristic.

### 6.1 Implications

Drawing on evidence from 15 randomized controlled trials, this review evaluated the extent to which physical activity interventions may be linked to academic performance in children and adolescents. The findings suggest that physical activity may be associated with small benefits in some academic domains, including mathematics and reading, while the evidence for overall academic performance should be interpreted more cautiously. These effects were not uniform across outcomes, and exploratory subgroup analyses suggested differing pooled patterns according to intervention type, frequency, session duration, and intervention period. However, these findings were based on a small number of studies for several comparisons and should be interpreted cautiously rather than as confirmatory evidence of between-subgroup differences.

These findings provide preliminary and cautious support for incorporating movement into school-based academic activities. Physical activity may benefit not only physical health but also educational practice. Future research should further clarify which intervention characteristics are associated with more favorable academic outcomes across different age groups, educational contexts, and academic domains.

## References

[ref1] Ardoy D. N. Fernández-Rodríguez J. M. Jiménez-Pavón D. Castillo R. Ruiz J. R. Ortega F. B. (2014). A physical education trial improves adolescents' cognitive performance and academic achievement: the EDUFIT study. Scand. J. Med. Sci. Sports 24, e52–e61. doi: 10.1111/sms.12093, 23826633

[ref2] Atakan M. M. Atakan B. (2024). Acute Pilates and plyometric exercise in school-based settings improve attention and mathematics performance in high school students. Sports Med. Health Sci. 6, 185–192. doi: 10.1016/j.smhs.2023.12.008, 38708323 PMC11067860

[ref3] Baker A. Galván A. Fuligni A. (2024). The connecting brain in context: how adolescent plasticity supports learning and development. Dev. Cogn. Neurosci. 71:101486. doi: 10.1016/j.dcn.2024.101486 39631105 PMC11653146

[ref4] Beck M. M. Lind R. R. Geertsen S. S. Ritz C. Lundbye-Jensen J. Wienecke J. (2016). Motor-enriched learning activities can improve mathematical performance in preadolescent children. Front. Hum. Neurosci. 10:645. doi: 10.3389/fnhum.2016.00645, 28066215 PMC5179540

[ref5] Berse T. Rolfes K. Barenberg J. Dutke S. Kuhlenbäumer G. Völker K. . (2015). Acute physical exercise improves shifting in adolescents at school: evidence for a dopaminergic contribution. Front. Behav. Neurosci. 9:196. doi: 10.3389/fnbeh.2015.00196, 26283937 PMC4517060

[ref6] Bodensohn L. Maurer A. Daamen M. Upadhyay N. Werkhausen J. Lohaus M. . (2024). Inverted U-shape-like functional connectivity alterations in cognitive resting-state networks depending on exercise intensity: an fMRI study. Brain Cogn. 177:106156. doi: 10.1016/j.bandc.2024.106156, 38613926

[ref7] Chandler J. Higgins J. P. T. Thomas J. Higgins J. P. T. Deeks J. J. Cochrane Handbook for systematic Reviews of Interventions. Hoboken: Wiley, (2019). 4: p. 14651858.

[ref8] Cipriano C. Barnes T. N. Rivers S. E. Brackett M. A. Elbertson N. A. . (2024). Physical activity interventions in educational settings: effects on academic achievement revisited. Int. Multidiscip. J. Res. Innov. Sustain. Excell.(IMJRISE) 1, 238–246. doi: 10.1007/s10648-010-9145-7

[ref9] Copenhagen: The Nordic Cochrane Centre; (2014). Review Manager (RevMan)[Computer program] Available online at: https://community.cochrane.org/help/tools-and-software/revman-5 (accessed 2019-03-15).

[ref10] Cumpston M. Li T. Page M. J. Chandler J. Welch V. A. . (2019). Updated guidance for trusted systematic reviews: a new edition of the Cochrane handbook for systematic reviews of interventions. Cochrane Database Syst. Rev. 2019:ED000142. doi: 10.1002/14651858.ed000142 PMC10284251 31643080

[ref11] De Bruijn A. G. M. Kostons D. D. N. M. Van Der Fels I. M. J. Visscher C. Oosterlaan J. Hartman E. . (2020). Effects of aerobic and cognitively-engaging physical activity on academic skills: a cluster randomized controlled trial. J. Sports Sci. 38, 1806–1817. doi: 10.1080/02640414.2020.1756680, 32567975

[ref12] De Greeff J. W. Bosker R. J. Oosterlaan J. Visscher C. Hartman E. . (2018). Effects of physical activity on executive functions, attention and academic performance in preadolescent children: a meta-analysis. J. Sci. Med. Sport 21:29054748, 501–507. doi: 10.1016/j.jsams.2017.09.595 29054748

[ref13] Donnelly J. E. Hillman C. H. Greene J. L. Hansen D. M. Gibson C. A. Sullivan D. K. . (2017). Physical activity and academic achievement across the curriculum: results from a 3-year cluster-randomized trial. Prev. Med. 99, 140–145. doi: 10.1016/j.ypmed.2017.02.006, 28193490 PMC6148354

[ref14] Drollette E. S. O’Brokta M. M. Pasupathi P. A. Cornwall A. S. Slutsky-Ganesh A. B. Etnier J. L. (2025). The effects of short exercise bouts on error-related negativity (ERN) and academic achievement in children. Psychol. Sport Exerc. 79:102847. doi: 10.1016/j.psychsport.2025.102847, 40157437

[ref15] Geary D. C. (2011). Consequences, characteristics, and causes of mathematical learning disabilities and persistent low achievement in mathematics. J. Dev. Behav. Pediatr. 32, 250–263. doi: 10.1097/dbp.0b013e318209edef 21285895 PMC3131082

[ref16] González-Pérez M. Grao-Cruces A. Bandera-Campos F. J. Chalkley A. Camiletti-Moirón D. Sánchez-Oliva D. (2025). Less is more: qualitative insights into physically active learning in secondary math education. PLoS One 20:e0336641. doi: 10.1371/journal.pone.0336641, 41252389 PMC12626303

[ref17] Gonzalez-Perez M. Sánchez-Oliva D. Martín-Acosta F. Ruiz-Hermosa A. Camiletti-Moirón D. Grao-Cruces A. (2025). A mixed-methods approach of the effect of physically active learning on time-on-task in the secondary education class: the ACTIVE CLASS study. Learn. Instr. 97:102091. doi: 10.1016/j.learninstruc.2025.102091

[ref18] He H. Yang Y. Sun J. Wang F. Zhang W. Zhu F. (2025). Effects of school-based physical activity on academic achievement in children and adolescents: a systematic review and meta-analysis. Front. Public Health 13:1651883. doi: 10.3389/fpubh.2025.1651883, 41036117 PMC12479424

[ref19] Hillman C. H. Pontifex M. B. Castelli D. M. Khan N. A. Raine L. B. Scudder M. R. . (2014). Effects of the FITKids randomized controlled trial on executive control and brain function. Pediatrics 134, e1063–e1071. doi: 10.1542/peds.2013-3219, 25266425 PMC4179093

[ref20] Hillman C. H. Pontifex M. B. Raine L. B. Castelli D. M. Hall E. E. Kramer A. F. (2009). The effect of acute treadmill walking on cognitive control and academic achievement in preadolescent children. Neuroscience 159, 1044–1054. doi: 10.1016/j.neuroscience.2009.01.057, 19356688 PMC2667807

[ref21] Hraste M. De Giorgio A. Jelaska P. M. Padulo J. Granić I. (2018). When mathematics meets physical activity in the school-aged child: the effect of an integrated motor and cognitive approach to learning geometry. PLoS One 13:e0196024. doi: 10.1371/journal.pone.0196024, 30089116 PMC6082508

[ref22] Janssen I. LeBlanc A. G. (2010). Systematic review of the health benefits of physical activity and fitness in school-aged children and youth. Int. J. Behav. Nutr. Phys. Act. 7:40. doi: 10.1186/1479-5868-7-40, 20459784 PMC2885312

[ref23] Jeppesen L. S. Damsgaard L. Stolpe M. N. Melcher J. N. S. Wienecke J. Nielsen G. . (2024). Study protocol for the Active School study investigating two different strategies of physical activity to improve academic performance in schoolchildren. BMC Pediatr. 24:174. doi: 10.1186/s12887-024-04647-9, 38461348 PMC10924402

[ref24] Joubert L. Kilgas M. Riley A. Gautam Y. Donath L. Drum S. (2017). In-class cycling to augment college student academic performance and reduce physical inactivity: results from an RCT. Int. J. Environ. Res. Public Health 14:1343. doi: 10.3390/ijerph14111343, 29113036 PMC5707982

[ref25] Li D. Wang D. Zou J. Li C. Qian H. Yan J. . (2023). Effect of physical activity interventions on children's academic performance: a systematic review and meta-analysis. Eur. J. Pediatr. 182, 3587–3601. doi: 10.1007/s00431-023-05009-w, 37227500

[ref26] Liu L. Xin X. Zhang Y. (2025). The effects of physical exercise on cognitive function in adolescents: a systematic review and meta-analysis. Front. Psychol. 16:1556721. doi: 10.3389/fpsyg.2025.1556721, 40792082 PMC12337486

[ref27] Loturco I. Montoya N. P. Ferraz M. B. Berbat V. Pereira L. A. (2022). A systematic review of the effects of physical activity on specific academic skills of school students. Educ. Sci. 12:134. doi: 10.3390/educsci12020134

[ref28] Lubans D. R. Smith J. J. Peralta L. R. Plotnikoff R. C. Okely A. D. . (2018). School physical activity intervention effect on adolescents' performance in mathematics. Med. Sci. Sports Exerc. 50:30067590, 2442–2450. doi: 10.1249/MSS.0000000000001730 30067590

[ref29] Lv Y. Dong X. Sun T. Jiang S. Gao Y. Liang J. . (2024). Acute effects of different physical activity on executive function and regulation role of beta oscillation in sedentary youth frontal region. Sci. Rep. 14:30939. doi: 10.1038/s41598-024-81538-0, 39730640 PMC11681212

[ref30] Maiztegi-Kortabarria J. Arribas-Galarraga S. de Luis- Cos I. Espoz-Lazo S. Valdivia-Moral P. (2024). Effect of an active break intervention on attention, concentration, academic performance, and self-concept in compulsory secondary education. Eur. J. Investig. Health Psychol. Educ. 14, 447–462. doi: 10.3390/ejihpe14030030, 38534891 PMC10969120

[ref31] Melero-Cañas D. Morales-Baños V. Ardoy D. N. Manzano-Sánchez D. Valero-Valenzuela A. (2021). Enhancements in cognitive performance and academic achievement in adolescents through the hybridization of an instructional model with gamification in physical education. Sustainability 13:5966. doi: 10.3390/su13115966

[ref32] Moher D. Liberati A. Tetzlaff J. Altman D. G. (2009). Preferred reporting items for systematic reviews and meta-analyses: the PRISMA statement. BMJ 339:b2535. doi: 10.1136/bmj.b2535, 19622551 PMC2714657

[ref33] Mullender-Wijnsma M. J. Hartman E. de Greeff J. W. Doolaard S. Bosker R. J. . (2016). Physically active math and language lessons improve academic achievement: a cluster randomized controlled trial. Pediatrics 137:e20152743. doi: 10.1542/peds.2015-2743 26912206

[ref34] Naito T. Oka K. Ishii K. (2024). Hemodynamics of short-duration light-intensity physical exercise in the prefrontal cortex of children: a functional near-infrared spectroscopy study. Sci. Rep. 14:15587. doi: 10.1038/s41598-024-66598-6, 38971930 PMC11227512

[ref35] Norris E. Shelton N. Dunsmuir S. Duke-Williams O. Stamatakis E. . (2020). Physically active lessons in schools and their impact on physical activity, educational, health and cognition outcomes: a systematic review and meta-analysis. Br. J. Sports Med. 54, 826–838. doi: 10.1136/bjsports-2018-100502 31619381

[ref36] Owen K. B. Parker P. D. Astell-Burt T. Lonsdale C. Dollman J. . (2023). Sport participation for academic success: evidence from the longitudinal study of Australian children. J. Phys. Act. Health 21, 238–246. doi: 10.1123/jpah.2023-0506 38141604

[ref37] Peternelj B. Škof B. Strel J. (2009). Academic achievement of pupils in sport classes: pupils attending sport classes have higher final grades, but…. Kinesiol. Slov. 15, 5–16.

[ref38] Resaland G. K. Aadland E. Moe V. F. Aadland K. N. Skrede T. Stavnsbo M. . (2016). Effects of physical activity on schoolchildren's academic performance: the active smarter kids (ASK) cluster-randomized controlled trial. Prev. Med. 91, 322–328. doi: 10.1016/j.ypmed.2016.09.005, 27612574

[ref39] Riley N. Lubans D. R. Holmes K. Morgan P. J. (2016). Findings from the EASY minds cluster randomized controlled trial: evaluation of a physical activity integration program for mathematics in primary schools. J. Phys. Act. Health 13, 198–206. doi: 10.1123/jpah.2015-0046, 26107532

[ref40] Sallis J. F. McKenzie T. L. Alcaraz J. E. Kolody B. Faucette N. Hovell M. F. (1997). The effects of a 2-year physical education program (SPARK) on physical activity and fitness in elementary school students. Sports, play and active recreation for kids. Am. J. Public Health 87, 1328–1334. doi: 10.2105/AJPH.87.8.1328, 9279269 PMC1381094

[ref41] Singh A. S. Saliasi E. van den Berg V. Uijtdewilligen L. de Groot R. H. M. Jolles J. . (2019). Effects of physical activity interventions on cognitive and academic performance in children and adolescents: a novel combination of a systematic review and recommendations from an expert panel. Br. J. Sports Med. 53, 640–647. doi: 10.1136/bjsports-2017-098136, 30061304

[ref42] Solberg R. B. Steene-Johannessen J. Anderssen S. A. Ekelund U. Säfvenbom R. Haugen T. . (2021a). Effects of a school-based physical activity intervention on academic performance in 14-year old adolescents: a cluster randomized controlled trial - the school in motion study. BMC Public Health 21:871. doi: 10.1186/s12889-021-10901-x, 33957895 PMC8101111

[ref43] Solberg R. B. Aadland E. Resaland G. K. Moe V. F. Steene-Johannessen J. . (2021b). Aerobic fitness mediates the intervention effects of a school-based physical activity intervention on academic performance. The school in motion study – a cluster randomized controlled trial. Prev. Med. Rep. 24:101566. doi: 10.1249/01.mss.0000876068.12516.81 PMC8684017 34976697

[ref44] Sterne J. A. Harbord R. M. (2004). Funnel plots in meta-analysis. Stata J. 4, 127–141. doi: 10.1177/1536867x0400400204

[ref45] Takehara K. Togoobaatar G. Kikuchi A. Lkhagvasuren G. Lkhagvasuren A. Aoki A. . (2021). Exercise intervention for academic achievement among children: a randomized controlled trial. Pediatrics 148:e2021052803. doi: 10.1542/peds.2021-052808, 34663681

[ref46] Tarp J. Domazet S. L. Froberg K. Hillman C. H. Andersen L. B. Bugge A. (2016). Effectiveness of a school-based physical activity intervention on cognitive performance in Danish adolescents: lcomotion—learning, cognition and motion–a cluster randomized controlled trial. PLoS One 11:e0158087. doi: 10.1371/journal.pone.0158087, 27341346 PMC4920412

[ref47] Unterguggenberger S. (2025). Physical activity, cognitive health and learning in youth: a narrative umbrella review. Int. J. Environ. Res. Public Health 23:11. doi: 10.3390/ijerph23010011, 41595805 PMC12841123

[ref48] Valkenborghs S. R. Noetel M. Hillman C. H. Nilsson M. Smith J. J. Ortega F. B. . (2019). The impact of physical activity on brain structure and function in youth: a systematic review. Pediatrics 144:e20184032. doi: 10.1542/peds.2018-4032, 31554668

[ref49] Vetter M. O’Connor H. O’Dwyer N. Orr R. (2018). Learning "math on the move": effectiveness of a combined numeracy and physical activity program for primary school children. J. Phys. Act. Health 15, 492–498. doi: 10.1123/jpah.2017-0234, 29580132

[ref50] Visier-Alfonso M. E. Sánchez-López M. Álvarez-Bueno C. Ruiz-Hermosa A. Nieto-López M. Martínez-Vizcaíno V. (2022). Mediators between physical activity and academic achievement: a systematic review. Scand. J. Med. Sci. Sports 32, 452–464. doi: 10.1111/sms.14107, 34837413

[ref51] Wang T. Guo C. (2022). Inverted U-shaped relationship between physical activity and academic achievement among Chinese adolescents: on the mediating role of physical and mental health. Int. J. Environ. Res. Public Health 19:4678. doi: 10.3390/ijerph19084678, 35457546 PMC9025370

[ref52] Wang D. C. Li Y. Chen S. Zhang Y. Li X. . (2023). Effect of extracurricular after-school physical activities on academic performance of schoolchildren a cluster randomized clinical trial. JAMA Pediatr. 177, 1141–1148. doi: 10.1001/jamapediatrics.2023.3615 37721735 PMC10507588

[ref53] Xu Y. Zhang X. Chen H. Liu Y. Wang L. . (2023). The effect of classroom-based physical activity elements on academic performance in children and adolescents: a meta-analysis. J. Teach. Phys. Educ. 43, 79–92. doi: 10.1123/jtpe.2023-0035

[ref54] Zheng G. Ye B. Zheng Y. Xiong Z. Xia R. Qiu P. . (2019). The effects of exercise on the structure of cognitive related brain regions: a meta-analysis of functional neuroimaging data. Int. J. Neurosci. 129, 406–415. doi: 10.1080/00207454.2018.1508135, 30073877

